# A New Laccase Based Biosensor for Tartrazine

**DOI:** 10.3390/s17122859

**Published:** 2017-12-09

**Authors:** Siti Zulaikha Mazlan, Yook Heng Lee, Sharina Abu Hanifah

**Affiliations:** 1 School of Chemical Sciences and Food Technology, Faculty of Science and Technology, Universiti Kebangsaan Malaysia, Bangi, Selangor 43600, Malaysia; ieykamazlan58@gmail.com (S.Z.M.); leeyookheng@yahoo.co.uk (Y.H.L.); 2 Polymer Research Center, Faculty of Science and Technology, Universiti Kebangsaan Malaysia, Bangi, Selangor 43600, Malaysia

**Keywords:** tartrazine, methacrylate-acrylate microspheres, AuNPs, electrochemical sensor, enzymatic biosensor

## Abstract

Laccase enzyme, a commonly used enzyme for the construction of biosensors for phenolic compounds was used for the first time to develop a new biosensor for the determination of the azo-dye tartrazine. The electrochemical biosensor was based on the immobilization of laccase on functionalized methacrylate-acrylate microspheres. The biosensor membrane is a composite of the laccase conjugated microspheres and gold nanoparticles (AuNPs) coated on a carbon-paste screen-printed electrode. The reaction involving tartrazine can be catalyzed by laccase enzyme, where the current change was measured by differential pulse voltammetry (DPV) at 1.1 V. The anodic peak current was linear within the tartrazine concentration range of 0.2 to 14 μM (*R*^2^ = 0.979) and the detection limit was 0.04 μM. Common food ingredients or additives such as glucose, sucrose, ascorbic acid, phenol and sunset yellow did not interfere with the biosensor response. Furthermore, the biosensor response was stable up to 30 days of storage period at 4 °C. Foods and beverage were used as real samples for the biosensor validation. The biosensor response to tartrazine showed no significant difference with a standard HPLC method for tartrazine analysis.

## 1. Introduction

Laccase (polyphenoloxidase; EC 1.10.3.2) is a well-known enzyme for the oxidization of a wide range of compounds such as polyphenols, methoxy-substituted phenols and diamines. This enzyme, which belongs to the blue multi-copper-oxidase family undergoes typical reactions where a phenolic group is often oxidised via one electron transfer to form a phenoxyl free radical. This results in an active oxygen species that could yield a quinone or further undergo polymerisation of the free radical. The ability of laccase to react with phenolic compounds opens up many opportunities of applications in agricultural, food, industrial, medical and environmental sectors [1].

Laccase based voltammetric/amperometric biosensors have been developed for food and beverages analyses. These biosensors have found wide applications in the analysis of phenol, polyphenol, guaiacol, gallic acid, caffeic acid, catechin, catechol, hydroquinone and resorcinol to name a few in various food and beverage products [2,3,4]. However, such biosensors have not been explored for food colour additives such as tartrazine. The selection of a suitable matrix for laccase enzyme immobilization in the construction of a biosensor for food analysis is a crucial part of the fabrication process [5]. The enzyme laccase immobilisation methods for the construction of biosensors reported for food analysis were based on several procedures involving laccase adsorptions (e.g., in carbon/graphite materials, nafion nanocomposite and screen-printed gold); enzyme entrapments (e.g., in polyazetidine prepolymer, chitosan, MWCNTs, PVA photopolymer and sonogel), enzyme cross-linkings (e.g., with cyanuric chloride/chitosan, polyvinylpropylidone gel, glutaradehyde on glassy carbon) and laccase covalent attachments (e.g., on nickel nanoparticles/MWCNTs/PANI, DEAE cellulose, ITO-APTES monolayer, glutaradehyde-cysteamine monolayer, magnetic nanoparticles or copper nanoparticles composite with MWCNTs and PANI) [6].

Polymeric microspheres are favourable materials for enzyme immobilization due to their high surface area in 3D shape, chemical stability, porosity and functional group density that can easily be tailored in accordance to their specific needs [7]. The poly(glycidyl methacrylate) (PGMA) containing epoxy group has been widely applied due to its attractive properties and discovered as an ideal support for enzyme immobilization [8]. They have the ability to form strong linkages with amino, hydroxyl, and thiols group under mild conditions [9,10]. The modifications of the epoxy ring with amine groups endow PGMA with excellent affinity to a variety of proteins, which makes it applicable in many areas, and easily available for immobilization of the enzyme [11]. The use of methacrylic based polymers in the forms of microspheres or cryogels for the immobilization of laccase have been reported with the aim of application to bioremediation and waste water treatment [12,13].

This study highlights the development of a new tartrazine biosensor based on laccase. To the best of our knowledge, detection of tartrazine catalyzed by laccase using the electrochemical biosensor technique has not yet to be reported. Tartrazine (Figure S1) is a synthetic organic food dye found in common food products such as beverages, candies, dairy products and bakery products [2]. However, the content of tartrazine must be controlled due to its potential harm to human beings, contributed from the azo groups (N=N) and aromatic ring structures [14]. In China, the permitted maximum limit of tartrazine additive in foods is 0.1 g/kg (individually or in combination [2]. Tartrazine will cause many adverse health effects such as allergies, migraines, eczema, anxiety, diarrhoea and childhood hyperactivity if they are excessively consumed [15]. Therefore, convenient, rapid and reliable methods for rapid determination of tartrazine are essential for the food safety assurance. Traditionally, tartrazine has been analysed using spectrophotometry [3], chromatography [4], mass spectrometry-chromatography [5], capillary electrophoresis [16] and electrochemical methods using various chemically modified electrodes [17].

In this work, laccase enzyme was immobilized on poly(glycidyl methacrylate-*co*-n-butyl acrylate) (poly(GMA-*co*-nBA)) microspheres, which we have reported recently [18]. Poly (n-butyl acrylate) has a hydrophobic property and thus the microspheres are hydrophobic where the laccase immobilization will be confined to the surface of the spheres; this allows the enzymatic reaction with tartrazine to occur at the surface and diffusion limitation within the polymer matrix is eliminated [7]. Thus, we have attempted for the first time to utilize laccase enzyme for the successful construction of a biosensor for tartrazine analysis.

## 2. Materials and Methods

### 2.1. Chemicals

Cyclic voltammetry (CV) and differential pulse voltammetry (DPV) experiments were performed with AutolabPGSTAT 12 (Autolab, Metrohm, Zofingen, Switzerland) potentiostat. The parameters used for CV were 0.007 V for step potential and 0.05 V/s of scan rate from −1.25 to 0.75 V. For DPV, the parameters used were 0.02 V step potential in the scan range of −1.0 to −0.1 V. A screen printed electrode (SPE) supplied by Scrint Technology (M) Sdn. Bhd. coated with methacrylate-acrylate microspheres in the presence of gold nanoparticles (AuNPs) was used as working electrode. A rod-shaped glassy carbon electrode and Ag/AgCl electrode were used as auxiliary and reference electrodes, respectively, and the KCl solution of 3.0 M was used as the internal solution of the Ag/AgCl electrode. All potentials measured in this study were referred to Ag/AgCl electrode and homogeneous mixture of material solutions was prepared using sonicator bath Elma S30H.

The following chemicals were obtained from commercial sources: glycidyl methacrylate, GMA (Sigma-Aldrich, St. Louis, MO, USA), n-butyl acrylate, nBA (Merck, Kenilworth, NJ, USA), ethylene glycol dimethacrylate, EGDMA (Sigma-Aldrich, St. Louis, MO, USA), sodium dodecyl sulphate, SDS (Systerm), 2,2-dimethoxy-2-phenylacetophenone, DMPP (Sigma-Aldrich, St. Louis, MO, USA), glutaric aldehyde, GA (Sigma-Aldrich, St. Louis, MO, USA), Bradford reagent (Sigma-Aldrich, St. Louis, MO, USA) and bovine serum albumin, BSA (Sigma-Aldrich, St. Louis, MO, USA). Deionized water was used for preparing aqueous solution during experiments.

### 2.2. Fabrication of Functionalized Microspheres and Electrochemical Characterization

Colloidal AuNPs (<100 nm particle size) was purchased commercially. About 1 g of methacrylate-acrylate microspheres were added to 10 mL enzyme solution and kept at 4 °C for 24 h in order to immobilize the enzyme onto the microspheres. The SPE working electrode (AuNPs/SPE) was prepared by depositing AuNPs onto SPE and dried at room temperature. Then, methacrylate-acrylate microspheres immobilized with laccase enzyme was suspended in 90:10 of ethanol to water before being deposited onto the AuNPs/SPE. It refers as microspheres-laccase/AuNPs/SPE and dried at room temperature. The response of tartrazine biosensor was later examined with CV and DPV in 0.05 μM phosphate buffer pH 5.0. Four SPEs were selected and fabricated including (a) bare SPE, (b) Laccase/SPE, (c) microspheres-laccase/SPE and (d) microspheres-laccase/AuNPs/SPE respectively. Electrochemical investigation of tartrazine was carried out in an electrochemical cell containing 4 mL of 0.1 M PBS (pH = 5.0) and 0.5 μM of TT. The potential range was 0.50 to 1.30 V using microspheres-laccase/AuNPs/SPE as working electrode, a glassy carbon counter electrode and Ag/AgCl reference electrode.

### 2.3. Optimization and Evaluation of Electrochemical Tartrazine Biosensor

The response of tartrazine biosensor was examined based on the effect of various parameters on the immobilized laccase. The influence of pH was analysed by varying pH of tartrazine solution that was prepared in 0.05 M sodium phosphate in the pH range 2.0–8.0 [19]. The exposure time, which is the duration of the exposure of the electrode to tartrazine, could affect the optimum biosensor response. For this study, the exposure time was performed from 1–15 min before measurement. The procedure was carried using a different amount of AuNPs between 0.02 mg and 0.10 mg. The microspheres amounts were also optimized. Effect of laccase concentration on biosensor response was also examined by varying laccase concentration over the range of 1.0–3.0 mg/mL. The voltammograms were recorded from +0.60 to +1.20 V at 0.07 V·s^−1^ scan rate. Under optimum conditions, DPV measurements were recorded from +0.60 to +1.20 V to obtain the linear range and low detection limit of tartrazine biosensor. Furthermore, to evaluate the interference that may interfere on the tartrazine determination, tartrazine concentration is fixed at 0.5 μM. Then, the interferences were added with various ratios of concentration. Other performances of biosensor including reproducibility and repeatability were also examined using DPV. Stability studies were carried out by storing electrode at 4 °C for 90 days. The electrode was immersed in 0.1 M PBS (pH = 5.0) when it was not in use. The electrode performance was checked daily using DPV [20,21].

### 2.4. Determination of Tartrazine in Food Samples

The samples were purchased from a local market. All simultaneous determinations of each sample were performed by standard addition method and have been repeated 3 times under the same conditions to examine and to improve the results’ accuracy. For electrochemical analysis, 20.0 mL commercial mango juice or 20.0 g candy coated chocolate was taken and dissolved in 20 mL 0.1 M PBS (pH 5.0). The synthetic dye content in commercial mango juice was determined by the standard addition method to prevent any matrix influence.

### 2.5. Validation and Recovery Studies of Tartrazine in Food Samples

Samples were also validated and analysed by high performance liquid chromatography (HPLC). HPLC system consisted of a binary pump, a degasser, an automated injector, a column oven and an UV–vis detector (Agilent 1100 Series HPLC, Agilent Technologies, Massachusetts, USA). HPLC was also introduced to detect tartrazine in drinks according to Alves et al. [22]. The column was a C18 analytical column (4.6 mm × 250 mm × 5 μm). One mobile phase system was employed to accomplish a quick separation of the analysed dyes in samples. It contained methanol (solution A) and 0.08 mol·L^−1^ aqueous ammonium acetate (solution B). Prior to usage, the aqueous solution and methanol were further filtered through 0.45 μm membranes. A constant flow rate was 0.7 mL·min^−1^ and the injection column was 20 mL. After completing the chromatographic elution, the mobile phase was programmed to its initial condition within 5 min, while 10 min reconditioning time was set before next injection. The detection was performed at a wavelength of 417 nm for both sunset yellow and tartrazine with UV-vis spectra detector. Statistical analysis (Student *t*-test) was performed by Microsoft Excel datasheets for comparison of both techniques.

## 3. Results

### 3.1. Methacrylate-Acrylate Microspheres

Surface morphology of methacrylate-acrylate microspheres was characterized by SEM and as presented in Figure S2A. The micrographs revealed that the synthesized copolymers were in spherical shape. However, when nBA composition was increased from 10% to 20%, the microspheres tended to be more aggregated, as exhibited by the micrograph (Figure S2D). This may be explained via a higher quantity of nBA that leads to the merging of the monomer into larger droplets; hence, an increase in the size of the microspheres formed after photopolymerization. The increase of sticky properties is caused by nBA [23]. The particle size of microspheres were then determined in Figure S2E. Copolymer of GN91 obtained the highest percentage of microspheres diameter in the range of 0.1 to 2.0 μm for GN91 copolymer compared to 1.0 to 4.0 μm for GN82. As a result, the total surface area of reaction will be increased and improved enzyme binding onto the polymer microspheres will occur, as reported by Ling and Heng (2010).

### 3.2. Electrochemical Behaviours of Tartrazine

**Figure 1:**
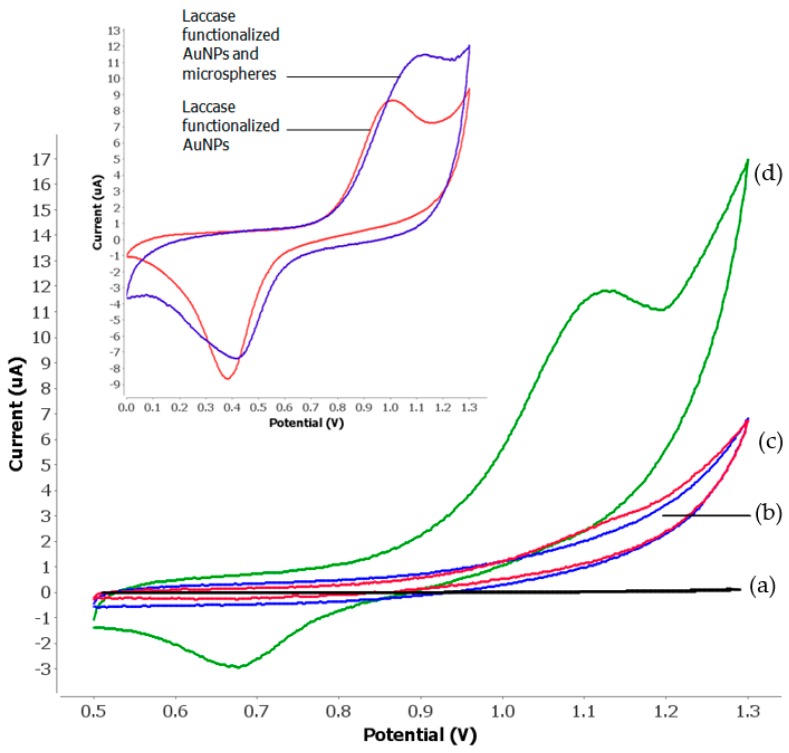
Cyclic voltammograms of (a) bare SPE, (b) Laccase/SPE, (c) microspheres-laccase/SPE and (d) microspheres-laccase/AuNPs/SPE in the presence of 0.5 μM Tartrazine in 0.1 M PBS pH 5.0. Scan rate: 0.07 V/s. A magnified view of the comparison between AuNP-only (red) and AuNP/microspheres (blue) electrodes voltammogram is shown at the inset.

Theoretically, tartrazine catalysed by laccase will produce a non-reversible reaction. As presented in Figure 1, there were different electrochemical behaviours revealed on (a) bare SPE (b) laccase/SPE, (c) microspheres-laccase/SPE and (d) microspheres-laccase/AuNPs/SPE. The electrochemical properties of tartrazine on the different carbon paste screen printed electrodes (SPE) were studied using cyclic voltammetry (CV). Figure 1 shows the electrochemical behaviour of 0.5 μM tartrazine. Bare SPE (a) confirmed that neither oxidation nor reduction peak was observed. In contrast, the oxidation peak of tartrazine increases significantly when both of the microspheres and AuNPs used simultaneously, as compared to 1(b) and 1(c). At the microspheres-laccase/SPE, tartrazine produced a small peak. This can be ascribed to poor conductivity and small effective surface area of reaction of modified electrode (curve c). Based on the inset voltammograms in Figure 1, the anodic peak current that refers to oxidation of tartrazine for electrode functionalized AuNPs and microspheres was much higher than the electrode without microspheres. This indicates that microspheres can increase the surface area of reaction in this system because many more enzymes could be immobilized on a larger surface area. It is well known that spheres have the largest surface to volume ratio so that in a small space, the area is the largest and this favours more enzyme immobilization.

This biosensor system needs microspheres since microspheres are covalently bound with laccase enzyme. Microspheres act as a surface of reaction for laccase and the analyte, tartrazine. Although the non-conductive polymeric microspheres were used, the electron transfer can happen via AuNPs towards the carbon SPE. The electron transfer depends strongly on the immobilization procedure. This is due to the distance between the prosthetic group of the enzyme and the fact that the electrode surface is often rather long for direct electron transfer [24]. To overcome its limitation that leads to the unfavourable conditions for electron transfer, an optimally designed electrode configuration has to ensure that the electron transfer distance between an immobilized enzyme and electrode surface is made as short as possible or conductive material such as AuNPs is added. AuNPs act as tunnels for electron transfer on SPE electrodes. The tunneling effect of AuNPs allow the electron with less energy to tunnel through the barrier and be detected by the transducer. These results prove that the SPE electrode surface coating with microspheres of methacrylate-acrylate-AuNPs can improve the efficiency of the electrode surface area of reaction (Sharma et al. 2008). After the introduction of methacrylate-acrylate microspheres-AuNPs on the SPE, tartrazine exhibited only an anodic peak at Figure 1d about +1.1 V, revealing that tartrazine underwent a totally irreversible process, while the cathodic peak at 0.67 V refers to oxidation of AuOH to Au [25]. At the microspheres-laccase/AuNPs/SPE (curve d), the peak current of tartrazine increased dramatically and the background was larger than that of microspheres-laccase/SPE, meaning that AuNPs can significantly enhance the current response.

**Scheme 1:**
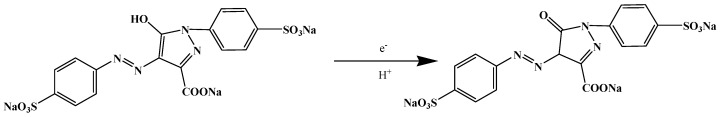
The proposed reaction of tartrazine detection using electrochemical technique. The oxidation occurs at 1.1 V under optimized condition.

Overall, tartrazine has an oxidation peak at 1.1 V. In the presence of the enzyme laccase, it helps to catalyze tartrazine to the oxidation state on the surface of the microspheres-laccase/AuNPs/SPE modified electrode through an electron transfer. Scheme 1 shows the proposed electrochemical process catalyzed by laccase.

### 3.3. Dependence of Biosensor Response on pH and Exposure Time

**Figure 2:**
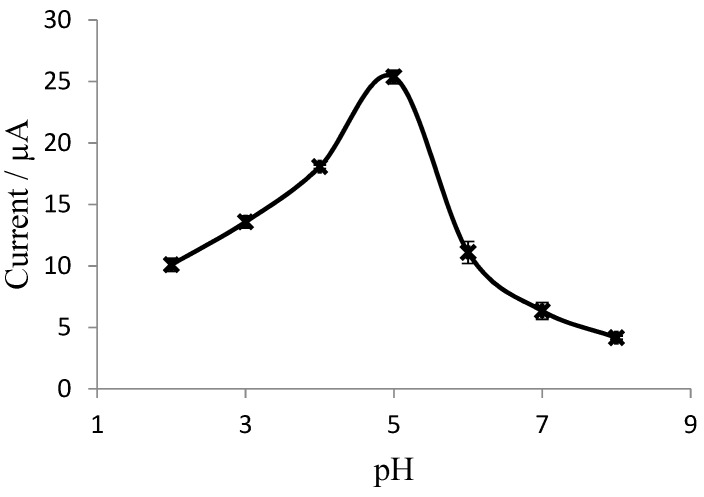
The effect of pH on the anodic peak potentials in 0.5 µM Tartrazine solution on microspheres-laccase/AuNPs/SPE. Exposure time: 2 min. Error bar represents the standard deviation of triple measurements.

The oxidation behaviours of tartrazine in buffer solutions with different pH values were studied to discuss the influences of pH value. As seen in Figure 2, the oxidation peak currents of Tartrazine on microspheres-laccase/AuNPs/SPE were gradually increased with pH value from 2.0 to 5.0, and then gradually decreased with further pH until pH 8. To obtain higher response signals, 0.1 M phosphate buffer with pH of 5.0 was used for the detection of tartrazine.

**Figure 3:**
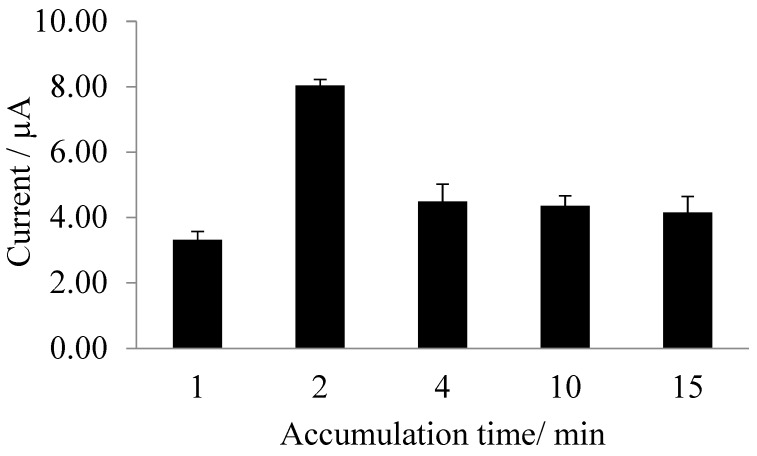
Effects of the exposure time on the oxidation peak current in the presence of 0.5 μM Tartrazine in 0.1 M PBS pH 5.0. Scan rate: 0.07 V/s. Error bar represents the standard deviation of triple measurements.

Figure 3 demonstrates the influences of exposure time of the electrode on the oxidation peak currents of Tartrazine. By extending the exposure time from 0 to 2 min, the signals of Tartrazine oxidation increased significantly, indicating that suitable exposure time had improved the detection sensitivity. However, their oxidation signals decreased again when the exposure time was longer than 2 min, which can probably be attributed to the deterioration of laccase and tartrazine reaction.

### 3.4. Effect of Microspheres and Nanogold Loading on Enzyme Biosensor Response

**Figure 4:**
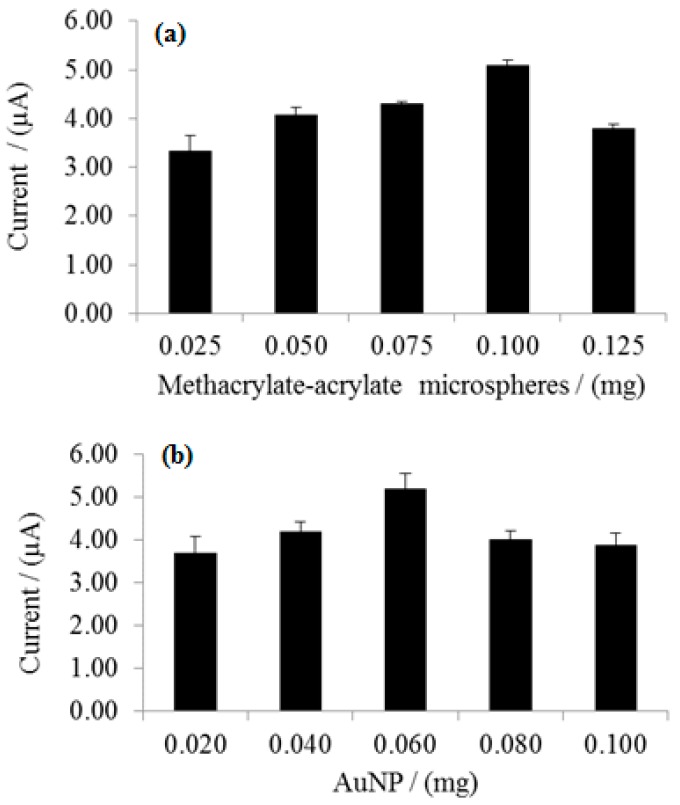
Anodic peak currents of 0.5 µM Tartrazine on the surface of modified electrode with various amounts of (**a**) methacrylate-acrylate microspheres and (**b**) quantity of AuNPs. Error bar represents the standard deviation of triple measurements.

The influence of methacrylate-acrylate microspheres on the voltammetry response of Tartrazine was explored. Results showed that the peak current increased quickly by increasing the amount of methacrylate-acrylate microspheres and reached the maximum response at 0.1 mg (Figure 4a), then further addition of methacrylate-acrylate microspheres caused a gradual decrease. The thickness of the working electrode will obviously increase the interface electron transfer resistance. Therefore, 0.1 mg of methacrylate-acrylate microspheres was adopted for further study.

AuNPs have significantly contributed in amplifying the response of tartrazine oxidation as shown in Figure 4b. When gradually increasing the amount of AuNPs from 0.020 to 0.060 mg, the oxidation peak currents of tartrazine enhance greatly; this is attributable to the electron transfer ability being improved. When more AuNPs were added, signals were decreased, possibly due to the electrode surface being covered by AuNPs. Thus, the analyte had difficulties penetratrating to the enzyme active site. Hence, conductivity of the system decreased. So, the optimum amount of AuNPs was 0.060 mg.

### 3.5. Influence of Laccase Immobilization Time

**Figure 5:**
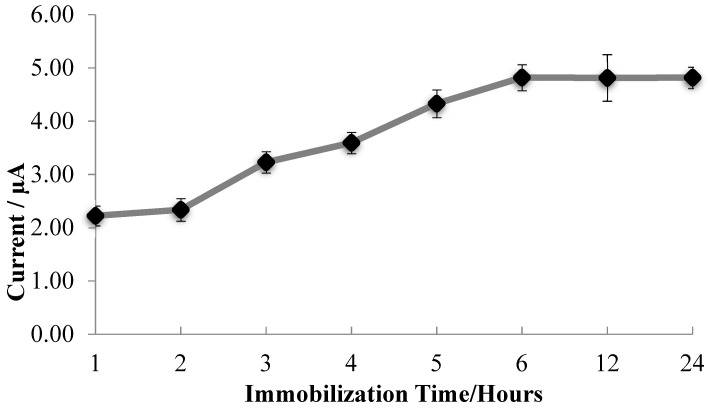
Influence of the laccase immobilization time on the anodic peak currents of 0.5 µM Tartrazine. Error bar represents the standard deviation of triple measurements.

Figure 5 shows a gradual increase of oxidation peak current from 1 h until 6 h. It then achieved equilibrium after 6 h. Longer immobilization time means more enzyme can attach with the microspheres. However, after optimum immobilization time (6 h), the signals were approximately stable, resulting from active sites of microspheres presumably being fully attached with laccase.

### 3.6. Linear Range and Detection Limit of Tartrazine Biosensor

**Figure 6:**
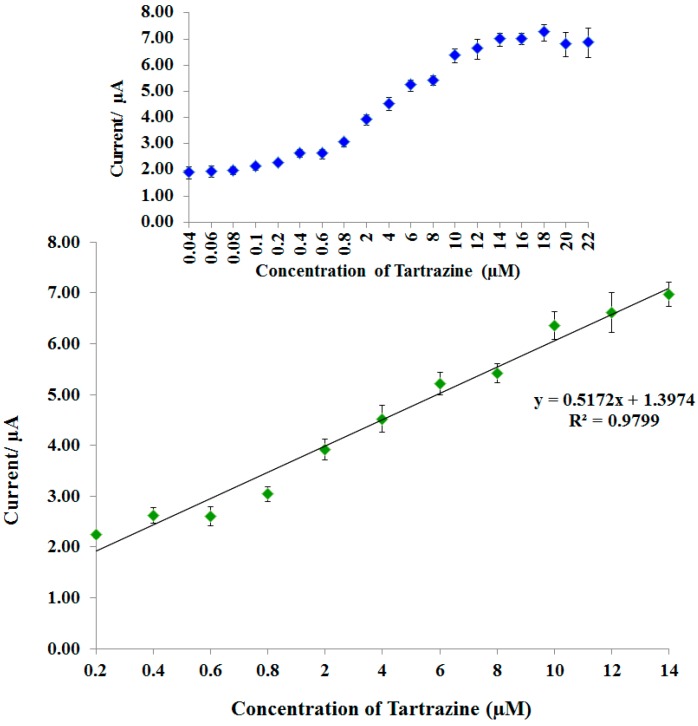
Calibration curve of the anodic peak currents as a function of Tartrazine concentrations in PBS (0.1 M, pH 5.0). Inset: Effect of concentration of Tartrazine solution. Accumulation time: 120 s; Error bar represents the standard deviation of triple measurements.

Electrochemical response of tartrazine on microspheres-laccase/AuNPs/SPE was examined using DPV in pH 5.0 phosphate buffer solution under the optimized experimental conditions. As shown in Figure 6, it is found that the oxidation current increased linearly with tartrazine concentration. The calibration curve of tartrazine response is *y* = 0.5172*x* + 1.3974 with correlation coefficient *R*^2^ = 0.9799 confirms good linearity and detection limit was 0.04 μM (*n* = 3).

The influence of common coexisting substances in tartrazine determinations, e.g., glucose, sucrose, ascorbic acid, phenol and the dye sunset yellow was investigated by DPV under optimized conditions. The microspheres-laccase/AuNPs electrode was evaluated by testing the electrochemical response of 0.5 µM tartrazine in the presence of various ratios (1:0.01, 1:0.1, 1:1, 1:10, 1:100) of these substances. The tolerance limit was taken as the maximum concentration of these substances, which caused an approximately ±10% relative error in the determination [6]. Peak currents showed small change below ±10%, which indicated that these substances had little interference effect on the microspheres-laccase/AuNPs/SPE based electrode and this confirmed a good biosensor selectivity as shown in Table 1. Most of laccase-based biosensors were developed for phenolic functionality at a different operating potential 0.8 V. Thus, other phenolic compounds are not expected to interfere with tartrazine measurements.

To evaluate the reproducibility, five microspheres-laccase/AuNPs/SPE modified electrodes were prepared in the same way and 5.0 µM and 0.08 µM of tartrazine solution was determined. As a result, the relative standard deviation (RSD) of the peak current was 2.37 % and 8.54 % (*n* = 5) respectively. The repeatability was investigated by monitoring a 5 µM tartrazine solution using one electrode deposited with the functionalized polymer microspheres immobilized with laccase and the RSD of peak current was 86.30 % (*n* = 5). As expected due to a non-reversible reaction of tartrazine, the electrode cannot be reused and is identified as a disposable biosensor.

Moreover, storage stability was investigated by measuring a tartrazine solution intermittently for 90 days. After the sensor was stored for 10 days at 4 °C, microspheres-laccase/AuNPs/SPE could retain 95.50% of tartrazine of its initial current response for 5.0 µM of tartrazine solution. After one month-storage, the response still retained 81.33% of its initial current response. Finally, the biosensor response still retained 51.1% of the initial response after 90 days. Stability results for this biosensor were comparable with others laccase based biosensors [20,21]. The results reflected good reproducibility and stability of microspheres-laccase/AuNPs/SPE modified electrode suggesting acceptable storage stability.

### 3.7. Validation of Tartrazine Biosensor in Food Samples

Finally, the microspheres-laccase/AuNPs/SPE was applied to the determination of tartrazine in food and drink samples, specifically candy coated with chocolate and commercial mango juice. The percentage of recovery was between 94.75 ± 5.10% and 108.00 ± 4.88% (*n* = 3) and the RSD was below 5%. This suggested that the method had good accuracy and reliability thus confirming that the detection of tartrazine in real samples can be carried out. The response from the biosensor method was compared with the HPLC method [26]. Table 2 compares the results of the analysis. The results obtained using the biosensor method was in good agreement with the HPLC method indicating that the newly-developed sensing material is reliable and validated.

Statistical analysis was performed to determine whether the developed method biosensor are correlated or not. These results showed that the biosensor method has a good correlation with the HPLC method with slopes of 0.9958 and 1.0577 respectively for candy coated with chocolate and commercial mango juice. In addition, *R*^2^ values 0.9958 and 0.9998 were obtained for both samples. The statistical comparisons of the obtained values by these methods for the determination of tartrazine were analyzed by student’s *t*-test. As can be seen from Table 2, the experimental *t*-values at *p* = 0.05 for both techniques did not exceed the theoretical ones. The results confirmed that the response measures by the biosensor method and the HPLC method were not different.

In comparison to many reported electrochemical sensors without using enzyme, the selectivity of the bioelectrode developed here towards tartrazine is much improved as many of the reported electrochemical sensors could not determine tartrazine alone when another dye sunset yellow is present. These chemical sensors would determine both of these food dyes together [27,28,29]. Apart from demonstrating the advantage on a good selectivity towards tartrazine, the biosensor developed here also shows similar response range (0.2 to 14 μM) when compared to electrochemical sensors reported for tartrazine [30,31].

## 4. Conclusions

A new electrochemical enzymatic biosensor based on laccase enzyme for tartrazine analysis was successfully developed. This new biosensor for tartrazine was fabricated from the immobilization of laccase on methacrylate-acrylate microspheres and composite with AuNPs, which then coated onto a SPE. A significant increase in the biosensor response upon detection of tartrazine was observed at 1.1 V and the signal was enhanced in the presence of gold nanoparticles (AuNPs). Furthermore, the modified electrode benefited wide linear range from 0.2 µM to 0.10 µM (*R*^2^ = 0.979) and the limit of detection (LOD) of 0.04 µM. Although the operation potential of the biosensor was at the high end, there was no significant interference to biosensor response observed from possible interference species or when applied to the analysis of tartrazine in real food samples. Thus, the biosensor demonstrated good selectivity towards tartrazine analyte. The biosensor was characterized by a relatively fast response in two minutes and it showed excellent selectivity for tartrazine determination in real samples.

## Figures and Tables

**Table 1:** Percent of interfering substance in enzymatic biosensor.

Ratios	Percent of Interference (%)				
	Glucose	Sucrose	Ascorbic Acid	Phenol	Sunset Yellow
1:0.01	−0.014	0.393	0.232	−0.043	−0.368
1:0.1	0.123	0.363	0.504	0.045	0.285
1:1	0.772	1.907	0.808	0.207	0.615
1:10	0.158	1.616	0.624	0.194	0.458
1:100	0.340	1.022	−0.592	−0.002	0.325

**Table 2:** Determination of tartrazine in candy coated chocolate and commercial mango juice samples by microspheres-laccase/AuNPs/SPE modified electrodes (*n* = 3).

Samples	Spiked (μM)	Biosensor (μM)	HPLC (μM)	Recovery (%)
Candy coated with chocolate	2	2.16	2.07	108.00
	4	3.79	3.93	94.75
	6	6.11	6.03	101.83
Commercial mango juice	2	1.91	1.86	95.50
	4	4.09	4.12	102.25
	6	5.87	6.06	97.83

## Supplementary Materials

The following are available online at www.mdpi.com/1424-8220/17/12/2859/s1, Figure S1: Chemical structure of tartrazine, Figure S2: SEM micrographs of polyGMA (A), polynBA (B), GN91 copolymer (C), GN82 copolymer (D) at 2.00 K magnification and (E) Size distribution of poly(GMA-*co*-nBA) microspheres in various composition.
