# Supplementary material for: A New Laccase Based Biosensor for Tartrazine
Source: Sensors (Basel). 2017 Dec 9;17(12):2859. doi: 10.3390/s17122859 (PMC5750762; doi:10.3390/s17122859)
Supplement: Supplementary file 1 [file sensors-17-02859-s001.pdf]

Supplementary data
A new laccase based biosensor for Tartrazine

Siti Zulaikha Mazlan[1], Lee Yook Heng[1], Sharina Abu Hanifah[1, 2*]

[1] School of Chemical Sciences and Food Technology, Faculty of Science and Technology, 43600 UniversitiKebangsaan Malaysia, Bangi, Selangor Malaysia.

[2] b Polymer Research Center, Faculty of Science and Technology 43600 Universiti Kebangsaan Malaysia, Bangi, Selangor Malaysia.

* Correspondence: sharina@ukm.edu.my.; Tel.: +603-8921 3902

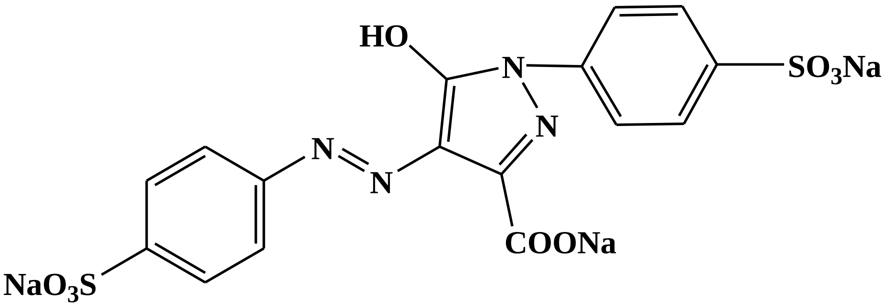

Fig. S1. Chemical structure of tartrazine

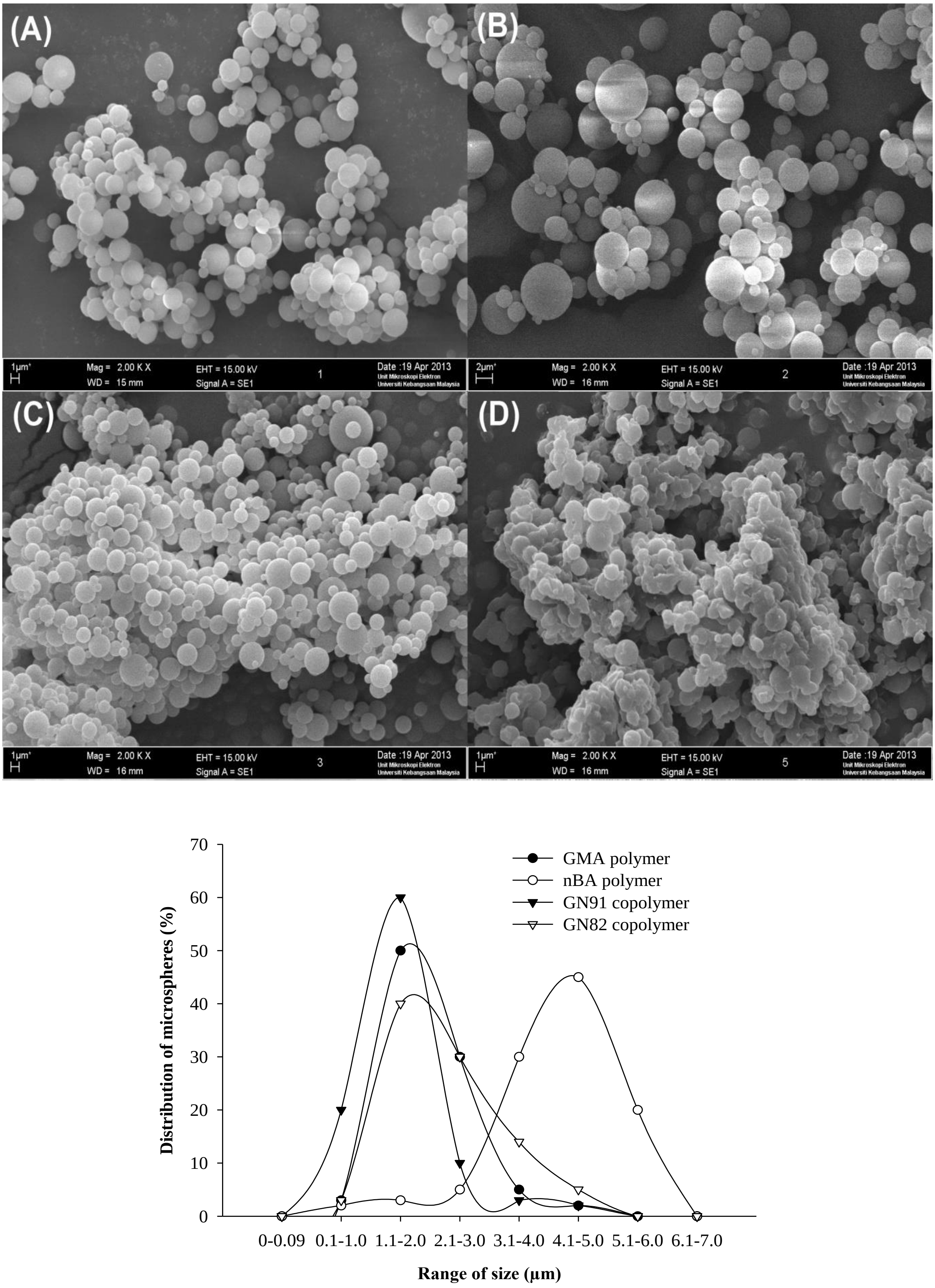

Fig. S2. SEM micrographs of polyGMA (A), poly*n*BA (B), GN91 copolymer (C), GN82 copolymer (D) at 2.00 K× magnification and (E) Size distribution of poly(GMA-co-*n*BA) microspheres in various composition.
